# Quantitative MRI Uncovers Subtle Cortical Damage in Myelin Oligodendrocyte Glycoprotein Antibody‐Associated Disease

**DOI:** 10.1002/acn3.70469

**Published:** 2026-07-13

**Authors:** Valentina Camera, Agnese Tamanti, Silvia Messina, Nicola Dall'Osto, Teresa Maltempo, Stefano Ziccardi, Matteo Foschi, Maria Grazia Piscaglia, Diana Ferraro, Francesco Crescenzo, Francesca Rossi, Albulena Bajrami, Sabrina Marangoni, Damiano Marastoni, Francesca Benedetta Pizzini, Maria Isabel Leite, Roberta Magliozzi, Patrick Waters, Massimiliano Calabrese, Jacqueline Palace, Ruth Geraldes

**Affiliations:** ^1^ Department of Neuroscience, Biomedicine and Movement Sciences University of Verona Verona Italy; ^2^ Nuffield Department of Clinical Neurosciences University of Oxford Oxford UK; ^3^ Department of Clinical Neurology, John Radcliffe Hospital Oxford University Hospitals Foundation Trust Oxford UK; ^4^ Department of Engineering for Innovation Medicine University of Verona Verona Italy; ^5^ Santa Maria Delle Croci Hospital, Department of Neuroscience MS Center Ravenna Italy; ^6^ Department of Biotechnological and Applied Clinical Sciences University of L'Aquila L'Aquila Italy; ^7^ Department of Neurosciences Azienda Ospedaliero‐Universitaria di Modena, Baggiovara Civil Hospital Modena Italy; ^8^ Neurology Unit Mater Salutis Hospital Legnago VR Italy; ^9^ Neurology Unit Santa Chiara Hospital Trento Italy; ^10^ Department of Brain Sciences, Faculty of Medicine Imperial College London London UK; ^11^ Oxford Autoimmune Neurology Diagnostic Laboratory, Nuffield Department of Clinical Neurosciences University of Oxford Oxford UK

**Keywords:** cognitive impairment, cortex, MOGAD, MTsat, quantitative MRI

## Abstract

**Objective:**

To determine whether myelin‐sensitive quantitative MRI reveals microstructural abnormalities in normal‐appearing cortex (NACtx) in myelin oligodendrocyte glycoprotein antibody–associated disease (MOGAD), indicating that conventional MRI underestimates remission residual cortical injury.

**Methods:**

Forty‐two patients with MOGAD in remission and 42 age‐ and sex‐matched healthy controls (HCs) underwent cognitive testing (Rao Brief Repeatable Battery), disability rating (Expanded Disability Status Scale) and 3‐T MRI, including three‐dimensional T1‐weighted, T2‐weighted and double‐inversion‐recovery sequences to identify cortical lesions and delineate NACtx. T1‐ to T2‐weighted ratio z‐scores (T1/T2r), magnetisation transfer ratio (MTR) and magnetisation transfer saturation (MTsat) were derived in global and lobar NACtx; MT imaging was available in 22 patients and 24 controls. Linear mixed‐effects models compared MOGAD with HCs and cortical (history of at least one acute cortical attack) with non‐cortical phenotypes, adjusting for age, sex, site and cortical thickness with false‐discovery‐rate correction.

**Results:**

Sixteen of 42 MOGAD patients had a cortical phenotype; cortical lesions at remission were present in 4 of 42 (9.5%), all with a cortical phenotype. MTsat metric, but not T1/T2r or MTR, detected NACtx alterations in the MOGAD cohort compared with HCs. Cortical MOGAD phenotype showed reduced MTsat in global, frontal, temporal, limbic, hippocampal and insular NACtx regions vs. HCs, whereas non‐cortical MOGAD was similar to HCs. Exploratory analyses suggested lower MTsat in temporal, limbic, hippocampal and insular NACtx regions in cortical MOGAD with cognitive impairment than in cognitively preserved MOGAD.

**Interpretation:**

Myelin‐sensitive MTsat reveals persistent, regionally specific abnormalities in normal‐appearing cortex in cortical MOGAD and is a promising marker of residual cortical damage linked to cognitive dysfunction.

## 1 Introduction

In myelin oligodendrocyte glycoprotein antibody‐associated disease (MOGAD), cortical involvement during acute attacks is frequent and may present as acute disseminated encephalomyelitis (ADEM), cerebral cortical encephalitis (CCE) or mono−/polyfocal cortical–subcortical syndromes [1, 2, 3, 4, 5, 6]. Histopathology from severe attacks shows predominantly perivenous cortical–subcortical demyelination with numerous small intracortical lesions and less frequent subpial and leukocortical lesions [7, 8].

In remission, however, cortical lesions are rarely detectable on conventional MRI or double inversion recovery (DIR) sequences [9, 10, 11], raising the question of whether apparent radiological normalisation reflects true cortical recovery or limited sensitivity of these techniques. Volumetric MRI studies indicate persistent cortical neuroaxonal injury, with reduced cortical volumes indicating residual structural damage, but how this relates to the acute cortical clinical phenotype remains unclear [12, 13, 14].

Advanced quantitative MRI methods that are sensitive to myelin and neurite architecture can uncover cortical microstructural abnormalities in tissue that appears normal on conventional imaging [15]. Magnetisation transfer imaging (MTI) provides an indirect measure of macromolecular myelin and neurite content by quantifying magnetisation exchange between free water and macromolecule‐bound protons [16]. The magnetisation transfer ratio (MTR), extensively validated in post‐mortem multiple sclerosis (MS) tissue as a marker of myelin integrity, axonal density, astrocytic proliferation and mitochondrial dysfunction [17, 18], is nevertheless influenced by field inhomogeneities and T1 relaxation, which can confound interpretation [16]. Magnetisation transfer saturation (MTsat) corrects for these confounds and yields more reliable myelin‐sensitive maps with improved tissue contrast compared with MTR [15, 16]. In parallel, the T1‐weighted/T2‐weighted ratio (T1/T2r), derived from routinely acquired sequences, has emerged as a marker of cortical microstructural integrity, with histopathological and imaging data in MS suggesting preferential sensitivity to neurite and dendritic density rather than demyelination [19]. As cortical macromolecular loss due to demyelination and neuroaxonal degeneration occurs, lower values are therefore expected for T1/T2r, MTR and MTsat [15, 16, 19]. Despite these advances, MOGAD‐related cortical tissue damage has been much less thoroughly characterised using advanced quantitative MRI.

On this background, the hypothesis was that microstructural cortical damage in MOGAD, particularly in patients with a history of acute cortical attacks (cortical MOGAD phenotype), is under‐recognised on conventional MRI and DIR in remission but can be detected using advanced quantitative MRI. The primary aim of this study was to assess the integrity of normal‐appearing cortex in MOGAD using the T1w/T2w ratio, MTR, and MTsat, compared with HCs. Secondary aims were to compare normal‐appearing cortical integrity between cortical and non‐cortical MOGAD phenotypes and to explore associations between cortical advanced quantitative imaging metrics and cognitive and physical outcomes.

## 2 Methods

### 2.1 Study Participants

This cross‐sectional study was conducted at the National Health Service (NHS) England–commissioned Neuromyelitis Optica Service in Oxford, United Kingdom, and at the Verona Multiple Sclerosis tertiary centre, Italy. Patients (≥ 16 years) with MOGAD fulfilling the 2023 international diagnostic criteria, confirmed by live cell‐based assays [1], were eligible if they were clinically and radiologically inactive for at least six months and were either untreated or receiving a stable disease‐modifying therapy (azathioprine, mycophenolate, methotrexate, or rituximab for at least 6 months, or low‐dose oral steroids for at least 3 months after initiation of tapering). Healthy controls (HCs) aged ≥ 16 years were enrolled, and all participants were required to be free from comorbidities likely to affect cognitive performance and to have no contraindications to MRI.

A total of 42 patients with MOGAD (Oxford, *n* = 20; Verona, *n* = 22) and 42 HCs (Oxford, *n* = 18; Verona, *n* = 24) were included. HCs were frequency‐matched to patients according to the group distributions of age and sex.

Clinical disability was assessed with the Expanded Disability Status Scale (EDSS), and cognition was evaluated using Rao's Brief Repeatable Battery of Neuropsychological Tests (BRB‐NT).

Patients with MOGAD were classified as having a “cortical MOGAD” or “non‐cortical MOGAD” phenotype. The cortical MOGAD phenotype was defined by a clinical history of cortical involvement in at least one prior attack, characterised by acute cortical inflammation with neurological features (e.g., focal seizures, aphasia, cortical motor‐sensory deficits, neglect or encephalopathy) typically supported, but not solely determined, by ancillary findings including cortical inflammatory MRI abnormalities and electrophysiological evidence of encephalic dysfunction.

Each MOGAD phenotype group was further subdivided according to cognitive status and disability. Cognitive impairment was defined as failure on at least one of five BRB‐NT tests (z‐score ≤ −1.5 standard deviations), whereas cognitively preserved status required no failed tests and all z‐scores > −1.5 standard deviations, using normative data from British [20] and Italian healthy populations [21]. Participants were additionally classified into two disability groups: Moderate‐to‐severe disability (EDSS ≥ 3) and no or minimal disability (EDSS < 3).

### 2.2 MRI Acquisition and Processing

MRI was performed on 3 T scanners: Siemens MAGNETOM Prisma (Oxford) and Philips Ingenia Elition‐S (Verona), using 64‐ and 32‐channel head coils, respectively. Acquisition protocols are detailed in eTable 1. 3D T1‐weighted (T1w), 3D/2D T2‐weighted (T2w), and 3D DIR sequences were acquired for all participants; MTI was available for a subgroup of 22 MOGAD patients and 24 HCs, all from the Verona Centre. T1/T2r ratio maps were generated using co‐registered intensity‐calibrated T1w and T2w images, with non‐brain tissue masks (eye and temporalis muscle) applied for calibration. Ratios were computed following the Ganzetti et al. pipeline [22], implemented by adapting the github.com/petergoodin/myelin_map released code, and converted to z‐scores using site‐specific HCs means and standard deviations. MTR was calculated from 3D Gradient Recalled Echo images with and without Magnetisation Transfer pulses [23]; MTsat was derived using an additional T1w acquisition, implemented in MATLAB R2022b [24].

### 2.3 Lesion Identification and Cortical Parcellation

Cortical–subcortical (leukocortical) lesions were identified on 3D DIR and 3DT1w images by consensus between two raters (VC, TM) and manually segmented using JIM v9.0, following Geurts et al. guidelines [25]. Cortical and subcortical parcellations were generated using multi‐atlas label fusion (MALF) [26], yielding global and lobar cortical masks (frontal, temporal, parietal, insular, limbic, occipital) and hippocampal regions. The final cortical lesion masks were derived from the overlap between the segmented lesion masks and the MALF‐derived masks of the cortical region of interest. Cortical lesion number was quantified, excluding lesions < 9 mm^3^. Cortical thickness was estimated from lesion‐filled 3DT1w images using FreeSurfer [27].

### 2.4 Quantitative MRI Analysis of Normal‐Appearing Cortex

**FIGURE 1:**
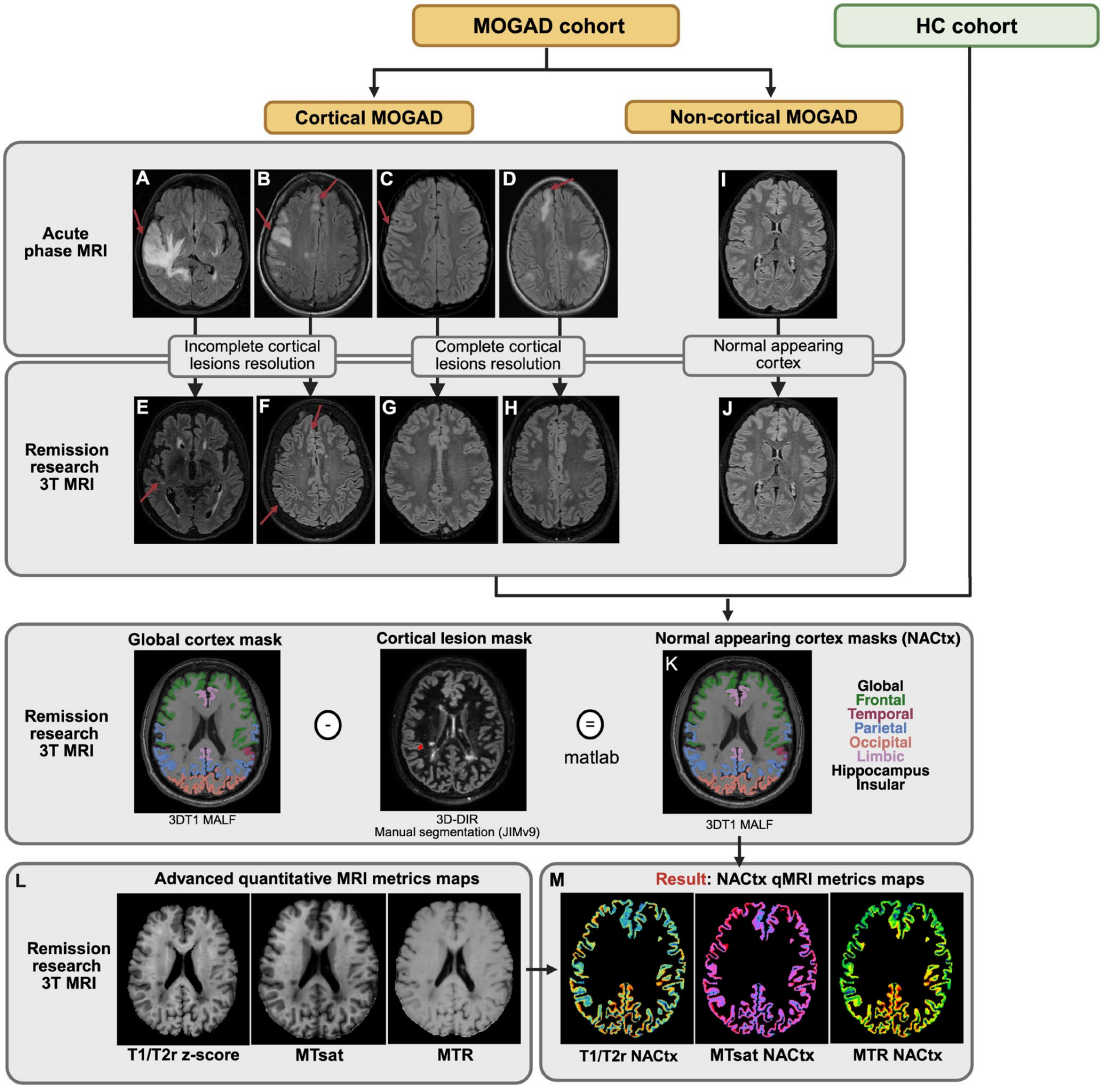
Simplified imaging methods pipeline. Top left: Exemplary cortical MOGAD acute clinical images: (A) ADEM with cortical involvement, encephalopathy, and status epilepticus; (B) ADEM without encephalopathy but with focal deficit and seizures; (C) right cerebral cortical encephalitis with status epilepticus; (D) isolated asymptomatic cortical lesions in a polyfocal syndrome. During remission, cortical lesions show incomplete (E, F) or complete (G, H) resolution on FLAIR. Top right: Non‐cortical MOGAD without cortical involvement during attacks (I) or remission (J). Centre: Global and lobar normal‐appearing cortex (NACtx) masks at remission MRI, obtained after removing cortical lesion masks from 3D T1‐weighted global cortex masks (K). Bottom: Overlap of myelin‐sensitive quantitative MRI (qMRI) maps (L) with global and lobar NACtx masks (M) to derive mean qMRI metrics in global and lobar NACtx. Created in https://biorender.com/.

Normal appearing cortex (NACtx) masks were derived by excluding dilated cortical lesion masks (2‐voxel radius) from MALF‐based cortical masks [28]. Mean qMRI values (T1/T2r, MTR, Mtsat) were computed for global and regional NACtx. T1/T2r values were converted to z‐scores to account for site‐specific distribution differences. Harmonisation of MTR and MTsat across sites was not required because MTI was performed exclusively in the Verona cohort. Figure 1 summarises the image processing methods pipeline.

### 2.5 Statistical Analysis

Normality of continuous variables was assessed with the Shapiro–Wilk test. Continuous data are presented as mean ± standard deviation (SD) or median (range), and categorical data as counts and percentages. Between‐group differences in normally distributed variables were evaluated with *t*‐tests or ANOVA, and with Kruskal–Wallis tests otherwise; categorical variables were compared using the chi‐squared test or Fisher's exact test, as appropriate.

For the primary analyses, linear mixed‐effects models were used to compare quantitative MRI metrics (T1/T2r z‐score, MTR, MTsat) in global and lobar NACtx masks between MOGAD and healthy controls (fixed effect), including MRI site (Oxford or Verona) as a random intercept. Models were first adjusted for age and sex (model 1), and then additionally for regional cortical thickness (model 2).

For the secondary analyses, analogous linear mixed‐effects models were fitted to compare quantitative MRI metrics between two‐level phenotypic groups (cortical MOGAD vs. non‐cortical MOGAD; cortical MOGAD vs. HCs; non‐cortical MOGAD vs. HCs; group as fixed effect) and to estimate differences in NACtx integrity between cortical MOGAD with cognitive impairment and cognitively preserved MOGAD. Multiple testing was controlled using the Benjamini–Hochberg false discovery rate (FDR) procedure, and FDR‐adjusted *p*‐values (p_adj) are reported.

In the cortical MOGAD subgroup, NACtx quantitative MRI metrics in global and lobar masks were explored descriptively, comparing patients with vs. without cognitive deficits and those with EDSS ≥ 3 vs. < 3; no formal hypothesis‐driven statistical testing was undertaken for these exploratory comparisons owing to the small sample size. All analyses were conducted in RStudio v1.4.110, with statistical significance predefined as *p* < 0.05 and 95% confidence intervals reported where applicable.

### 2.6 Standard Protocol Approvals, Registrations, and Patient Consents

Data were collected according to the NHS Research Ethics Committee protocols 16/SC/0224 and 17/EE/0246 for the Oxford cohort and according to the Southwest Veneto Territorial Ethic Committee (CET‐ASOV) protocol 2413CESC for the Verona cohort. All patients provided written consent for inclusion of their anonymised data and MR images.

## 3 Results

### 3.1 Demographic and Clinical Characteristics

**FIGURE 2:**
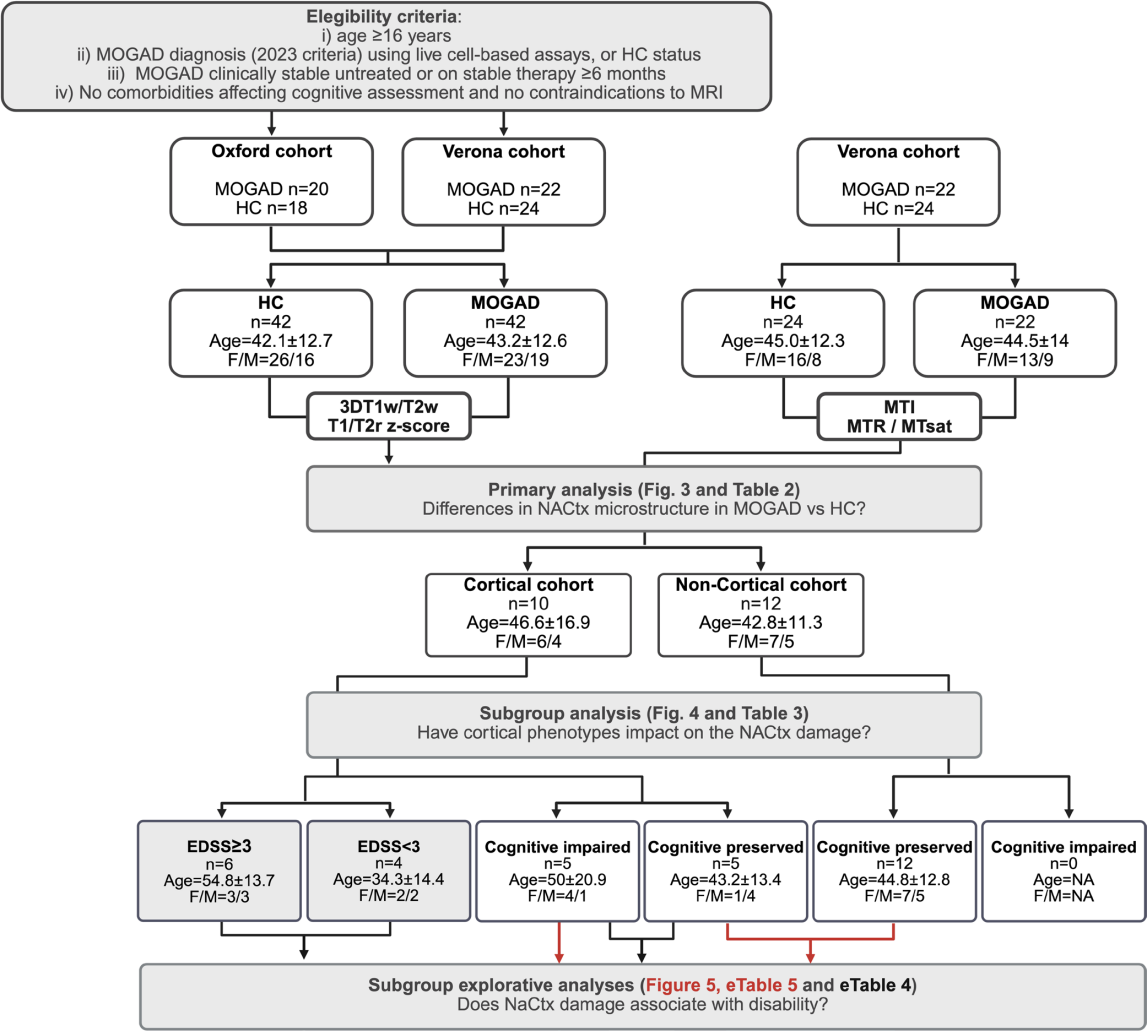
Study design to address the primary and secondary research questions. MOGAD = myelin‐oligodendrocyte‐glycoprotein antibody‐associated disease; HC = healthy controls; NACtx = normal‐appearing cortex on conventional imaging (T1w, T2w, DIR); qMRI = quantitative magnetic resonance imaging metrics; MTI = magnetisation transfer imaging. Created in https://BioRender.com.

Table 1 summarises demographic and clinical characteristics of the overall MOGAD cohort, the cortical and non‐cortical MOGAD subgroups, and the HCs cohort at recruitment. Of the 42 individuals with MOGAD, 16 (38%) had a history of cortical involvement in at least one of the previous attacks (cortical MOGAD), whereas 26 (62%) had never had cortical involvement (non‐cortical MOGAD). A relapsing disease course was observed in 14/16 (87.5%) cortical MOGAD and in 14/26 (53.8%) non‐cortical MOGAD patients (*p* = 0.042). Only 1/14 relapsing cortical MOGAD patients experienced recurrence in the same cortical region; the remaining 13 involved extra‐cortical sites (optic neuritis, transverse myelitis, or brainstem). This pattern suggests limited regional relapse specificity in cortical MOGAD. The median interval from the last clinical attack to MRI in the overall MOGAD cohort was 22 months (range 6–90 months), with no significant difference between cortical and non‐cortical subgroups. In the cortical MOGAD group, 14/16 patients (87.5%) had experienced their last clinical attack at least 12 months before imaging. Seizures or epilepsy were reported in 4/16 (25%) cortical MOGAD patients and in 0/26 non‐cortical patients (*p* = 0.016). Fifteen cortical–subcortical (leukocortical) lesions were identified on DIR in 4 of 42 patients with MOGAD (9.5%), all within the cortical subgroup (4/16; 25%) and none within the non‐cortical subgroup (0/26; 0%; *p* = 0.016). Failure in at least one cognitive test was observed in 7/16 (44%) cortical MOGAD patients and in 3/26 (11.5%) non‐cortical MOGAD patients (*p* = 0.027); in the latter subgroup, cognitive impairment occurred in the context of previous inflammatory involvement of deep grey matter structures. Figure 2 summarises participants’ flow and allocation according to available MRI sequences, indicating how total, cortical and non‐cortical MOGAD patients and HCs contributed to the primary, secondary, and exploratory analyses.

**TABLE 1:** Demographical and clinical features of the Myelin‐Oligodendrocyte‐Glycoprotein Antibody Associated Disease (MOGAD) cohorts and Healthy Controls (HC) at recruitment.

	Total cohort MOGAD (*n* = 42)	Cortical MOGAD (*n* = 16)	Non‐Cortical MOGAD (*n* = 26)	HC (*n* = 42)
**Demographics**				
Age, mean ± SD	43.2 ± 12.6	43.1 ± 13.7	43.2 ± 11.3	42.1 ± 12.7
Sex (F/M)	23/19	8/8	15/11	26/16
**Clinical features**				
Disease course:				
‐Monophasic, *n* (%)	14 (33.3)	2 (12.5)	12 (46.2)	NA
‐Relapsing, *n* (%)	28 (66.7)	14 (87.5)^a^	14 (53.8)^a^	NA
**Past attacks phenotype, *n* (%)**				
≥ 1 optic neuritis	24 (57)	7 (43.8)	17 (65.4)	NA
≥ 1 myelitis	22 (52.4)	6 (37.5)	16 (61.5)	NA
≥ 1brainstem/cerebellar syndrome	8 (19)	6 (37.5)	2 (7.7)	NA
≥ 1ADEM (with encephalopathy)	7 (16.7)	7 (43.8)	0	NA
≥ 1 Mono/Polyfocal syndrome	5 (12)	2 (12.5)	3 (11.5)	NA
≥ 1 CCE	5 (12)	5 (31.3)	0	NA
Median time from last attack, months (range)	22 (6–90)	23.5 (6–71)	20 (6–90)	NA
≥ 2 CSF oligoclonal bands, *n* (%)	9 (21.4)	5 (31.3)	4 (15.4)	NA
Seizures/epilepsy history at MRI, *n* (%)	4 (9.52)	4 (25)^a^	0^a^	NA
Cognitive test failure, *n* (%)	10 (23.8)	7 (44)^a^	3^+^ (11.5)^a^	NA
EDSS, median (range)	2 (0–7)	2 (0–6.5)	1.5 (0–7)	NA
Immunotherapy, (n)	Azathioprine (1)	Azathioprine (0)	Azathioprine (1)	NA
	Mycophenolate (4)	Mycophenolate (2)	Mycophenolate (2)	
	Methotrexate (2)	Methotrexate (1)	Methotrexate (1)	
	Rituximab (5)	Rituximab (3)	Rituximab (2)	
	No (30)	No (10)	No (20)	
	Low dose steroids*(8)	Low dose steroids*(3)	Low dose steroids*(5)	

Abbreviations: MOGAD: myelin oligodendrocyte glycoprotein antibody‐associated disease; cortical MOGAD = MOGAD patients with at least one prior relapse, including cortical inflammation; HC: healthy controls; ADEM = acute disseminated encephalomyelitis; CCE = cerebral cortical encephalitis; SD: standard deviation; CSF = cerebrospinal fluid; EDSS: Expanded Disability Status Scale; NA = not applicable.

^a^ Cortical MOGAD vs. non‐cortical MOGAD phenotypes; *p*‐value < 0.05.

* Low‐dose steroids in addition to one of the above treatments.

^+^ Acute attacks involving deep grey matter structures.

### 3.2 Quantitative MRI Cortex Characterisation in MOGAD

#### 3.2.1 Microstructural Integrity of Normal‐Appearing Cortex in MOGAD Vs. HC

Baseline clinical characteristics, brain lesional MRI metrics and regional cortical thickness measures in participants who underwent either the T1/T2r imaging or the MTI protocols to derive qMRI metrics are reported in eTable 2.

**FIGURE 3:**
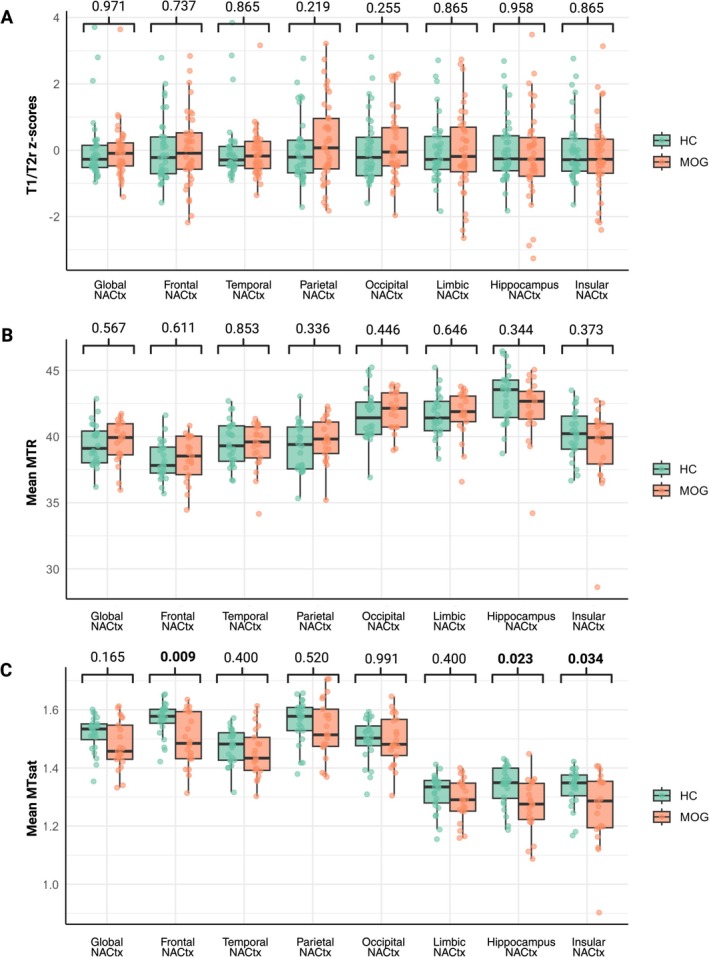
Regional normal‐appearing cortex quantitative MRI metrics in MOGAD vs. HC. Boxplots provide median and interquartile range of the mean qMRI metric (T1/T2r z‐score; MTR, MTsat) within the regional normal appearing cortices (NACTx) in MOGAD and HC. T1/T2r z‐score = T1‐weighted to T2‐weighted ratio z‐score; MTR = Magnetisation transfer ratio; MTsat = Magnetisation Transfer saturation. FDR‐corrected *p*‐value of linear mixed effect models 1 are provided dependent variable = mean regional NACtx qMRI metric; predictor variable = disease category (MOGAD vs. HC); covariates = age, sex; random effect = MRI scanner site (Verona vs. Oxford). p.u. = percentage units. Created in https://BioRender.com.

In both model 1 (adjusted for age and sex) and model 2 (additionally adjusted for cortical thickness), there were no group differences in T1/T2r z‐score or MTR in any region. No significant centre difference in T1/T2r z‐scores was observed between HC from Oxford (*n* = 18) and Verona (*n* = 24) in either model 1 or model 2 (all p_adj ≥ 0.514 and p_adj ≥ 0.606, respectively), confirming that the absence of T1/T2r differences between MOGAD and HC was not driven by site‐related effects. In contrast, compared with HC, patients with MOGAD showed lower MTsat in frontal (β −0.07; 95% CI −0.12 to −0.03; *p* = 0.002; p_adj = 0.009), hippocampal (β −0.07; 95% CI −0.11 to −0.02; *p* = 0.008; p_adj = 0.023), and insular (β −0.06; 95% CI −0.12 to −0.02; *p* = 0.012; p_adj = 0.034) NACtx. After additional adjustment for cortical thickness, effect directions were unchanged, with the hippocampal association remaining significant (p_adj = 0.034) and the frontal and insular associations attenuating (p_adj = 0.062 and 0.160, respectively) (Figure 3 and Table 2). In exploratory analyses, no clear differences were observed between treated and untreated MOGAD patients in cortical qMRI metrics, cortical thickness, or cognitive performance, although subgroup sizes were small and treatment categories heterogeneous.

**TABLE 2:** Mixed effect linear models comparing quantitative MRI metrics (T1/T2r, MTR, MTsat) in normal‐appearing cortex between MOGAD and HCs.

Region	T1/T2r z‐score MOGAD (*n* = 42) vs. HC (*n* = 42)		MTR MOGAD (*n* = 22) vs. HC (*n* = 24)		MTsat MOGAD (*n* = 22) vs. HC (*n* = 24)	
	Model 1 β [95% CI] *p*‐value (p _adj_)	Model 2 β [95% CI] *p*‐value (p _adj_)	Model 1 β [95% CI] *p*‐value (p _adj_)	Model 2 β [95% CI] *p*‐value (p _adj_)	Model 1 β [95% CI] *p*‐value (p _adj_)	Model 2 β [95% CI] *p*‐value (p _adj_)
**Global NACtx**	−0.007 [−0.37, 0.38]; 0.970 (0.971)	0.09 [−0.32, 0.50]; 0.677 (0.733)	0.31 [−0.60, 1.22]; 0.497 (0.567)	0.46 [−0.57, 1.51]; 0.369 (0.547)	−0.04 [−0.08, 0.003]; 0.067 (0.165)	−0.02 [−0.07, 0.025]; 0.343 (0.597)
**Frontal NACtx**	0.11 [−0.33, 0.56]; 0.621 (0.737)	0.18 [−0.31, 0.67]; 0.467 (0.620)	0.267 [−0.64, 1.17]; 0.555 (0.611)	0.36 [−0.62, 1.34]; 0.464 (0.619)	**−0.07 [−0.12, −0.03]; 0.002 (0.009)**	−0.06 [−0.11, −0.01]; 0.017 (0.062)
**Temporal NACTx**	−0.04 [−0.41, 0.33]; 0.838 (0.865)	−0.07 [−0.35, 0.48]; 0.748 (0.787)	−0.09 [−1.06, 0.88]; 0.853 (0.853)	0.37 [−1.72, 1.46]; 0.499 (0.624)	−0.03 [−0.07, 0.02]; 0.220 (0.400)	−0.03 [−0.08, 0.01]; 0.167 (0.351)
**Parietal NACtx**	0.33 [−0.13, 0.81]; 0.165 (0.219)	0.42 [−0.06, 0.90]; 0.089 (0.238)	0.67 [−0.37, 1.71]; 0.199 (0.336)	0.67 [−0.19, 1.87]; 0.199 (0.209)	−0.02 [−0.07, 0.03]; 0.389 (0.520)	−0.01 [−0.07, 0.04]; 0.646 (0.807)
**Occipital NACtx**	0.28 [−0.15, 0.72]; 0.207 (0.255)	0.27 [−0.18, 0.73]; 0.240 (0.400)	0.49 [−0.49, 1.47]; 0.321 (0.446)	0.76 [−0.27, 1.81]; 0.145 (0.254)	0.001 [−0.05, 0.05]; 0.988 (0.991)	0.03 [−0.01, 0.08]; 0.143 (0.322)
**Limbic NACtx**	0.06 [−0.44, 0.57]; 0.805 (0.865)	0.14 [−0.42, 0.69]; 0.632 (0.703)	0.25 [−0.73, 1.24]; 0.606 (0.646)	0.47 [−0.69, 1.63]; 0.421 (0.602)	−0.02 [−0.06, 0.02]; 0.225 (0.400)	−0.02 [−0.06, 0.03]; 0.514 (0.735)
**Hippocampus NACtx**	−0.06 [−0.57, 0.45]; 0.815 (0.958)	−0.06 [−0.57, 0.45]; 0.817 (0.838)	−0.80 [−2.08, 0.48]; 0.215 (0.344)	−0.77 [−2.00, 0.46]; 0.210 (0.330)	**−0.07 [−0.11, −0.02];** **0.008 (0.023)**	**−0.07 [−0.11, −0.02];** **0.008 (0.034)**
**Insular NACtx**	−0.07 [−0.53, 0.39]; 0.783 (0.865)	−0.18 [−0.67, 0.31]; 0.470 (0.619)	−0.78 [−2.11, 0.55]; 0.245 (0.373)	−0.48 [−0.19, 0.88]; 0.480 (0.619)	**−0.06 [−0.12, −0.02]; 0.012 (0.034)**	−0.05 [−0.12, 0.001]; 0.056 (0.160)

*Note:* Linear mixed effect models with mean MTsat in the NACtx region as the dependent variable, binary disease group (MOGAD vs. HC [reference]) as the primary predictor and random effect site (Oxford/Verona). Model 1 adjusted for age and sex. Model 2 adjusted for age, sex, and cortical thickness. β values report the between‐group difference with 95% CI and *p*‐value; the adjusted *p*‐value using FDR is shown in parentheses. Bold cells indicate results with adjusted *p*‐value < 0.05. MOGAD = myelin oligodendrocyte glycoprotein antibody‐associated disease; HC = healthy controls; NACtx = normal appearing cortex; T1/T2r z‐score = T1w‐toT2w signal ratio; MTR = Magnetization Transfer Ratio; MTsat = Magnetisation Transfer Saturation; CI = confidence interval; p _adj_ = *p*‐value corrected for multiple comparisons with false discovery rate.

As MTsat was the only quantitative MRI metric showing significant group differences, subsequent normal‐appearing cortical analyses were restricted to MOGAD and HC participants who underwent MTI.

#### 3.2.2 Cortical MOGAD Phenotype Drives Normal‐Appearing Cortex Microstructural Alterations

**FIGURE 4:**
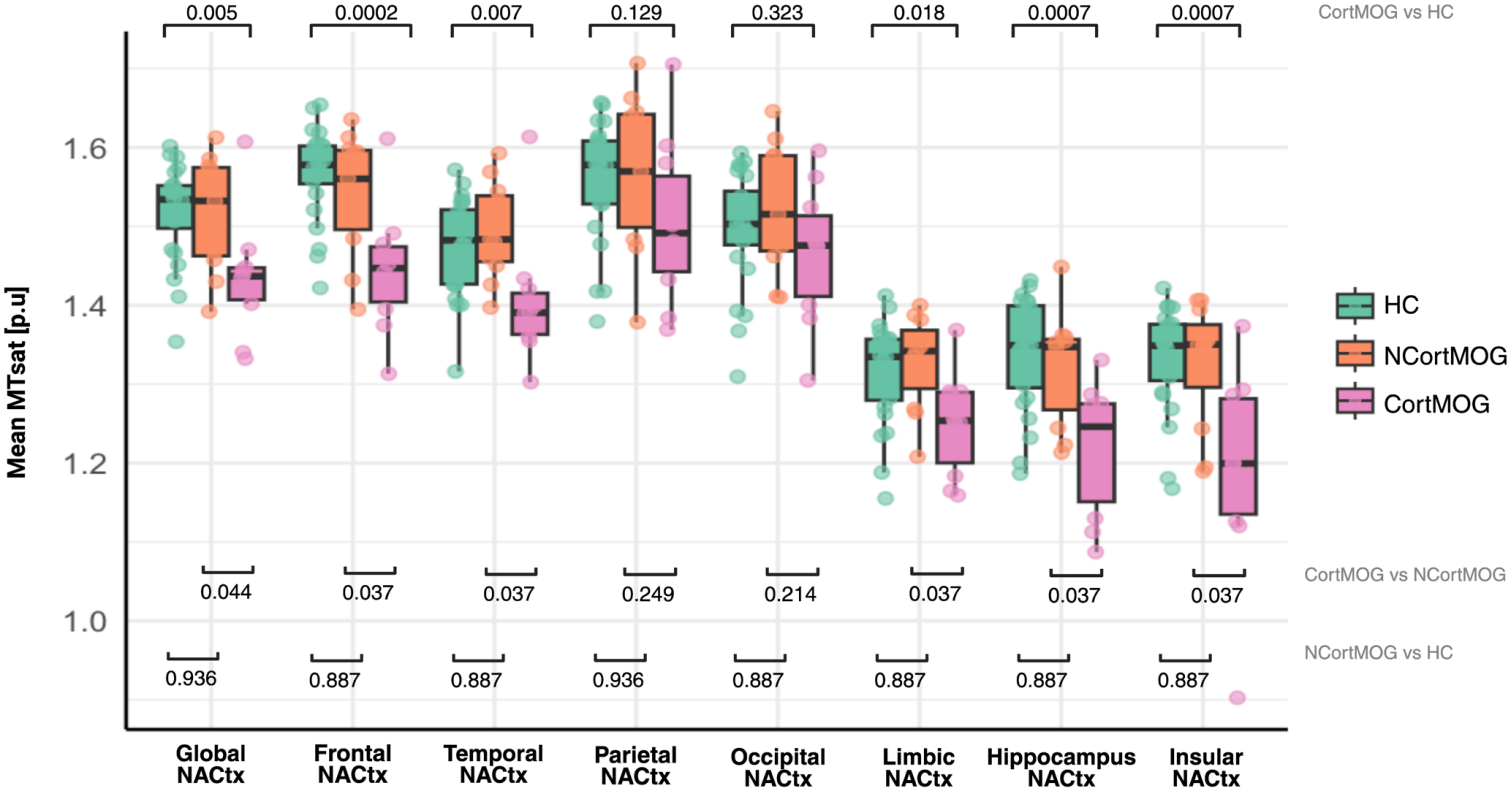
Regional normal‐appearing cortex MTsat in cortical, non‐cortical MOGAD phenotypes and healthy controls. Boxplots provide median and interquartile range of the mean MTsat within the regional normal appearing cortices (NACTx) in cortical (CortMOG), non‐cortical (NCortMOG) MOGAD and HC. FDR‐corrected *p*‐value of linear mixed effect models 1 are provided dependent variable = mean regional NACtx MTsat; predictor variable = binary disease phenotype (cortical MOGAD, non‐cortical MOGAD and HC); covariates = age, sex; random effect = MRI scanner site (Verona). p.u. = percentage units. Created in https://BioRender.com.

Clinical features, brain lesions and regional cortical thickness measures in the cortical and non‐cortical MOGAD subgroups and HCs with available MTsat metric are provided in eTable 3. Compared with the HCs cohort, patients with a cortical MOGAD phenotype showed significantly reduced MTsat within global (β = −0.08, 95% CI –0.14 to −0.03, *p* = 0.002; p_adj = 0.005), frontal (β = −0.12, 95% CI –0.17 to −0.07, *p* < 0.0001; p_adj = 0.0002), temporal (β = −0.08, 95% CI –0.13 to −0.03, *p* = 0.005; p_adj = 0.007), limbic (β = −0.07, 95% CI –0.12 to −0.01, *p* = 0.013; p_adj = 0.018), hippocampal (β = −0.12, 95% CI –0.18 to −0.06, *p* = 0.0002; p_adj = 0.0007), and insular (β = −0.13, 95% CI –0.19 to −0.07, *p* = 0.0002; p_adj = 0.0007) NACtx regions, with global, frontal, temporal, hippocampal, and insular effects persisting after additional adjustment for cortical thickness (Figure 4 and Table 3). In contrast, non‐cortical MOGAD cases did not show significant MTsat reductions relative to HCs in global or lobar NACtx. When directly compared, cortical MOGAD showed lower MTsat than non‐cortical MOGAD in global, frontal, temporal, limbic, hippocampal and insular NACtx, although these differences did not remain significant after further adjustment for cortical thickness (*p* = 0.051) (Figure 4 and Table 3).

**TABLE 3:** Linear mixed‐effects models comparing MTsat in normal‐appearing cortex across cortical MOGAD vs. non‐cortical MOGAD vs. HCs.

MTsat	Cortical MOGAD vs. HC (ref)		Cortical MOGAD vs. Non‐cortical MOGAD (ref)		Non‐cortical MOGAD vs. HC (ref)	
	Model 1 β [95% CI] *p*‐value (p _adj_)	Model 2 β [95% CI] *p*‐value (p _adj_)	Model 1 β [95% CI] *p*‐value (p _adj_)	Model 2 β [95% CI] *p*‐value (p _adj_)	Model 1 β [95% CI] *p*‐value (p _adj_)	Model 2 β [95% CI] *p*‐value (p _adj_)
**Global NACtx**	**−0.08 [−0.14, −0.03];** **0.002 (0.005)**	**−0.07 [−0.13, −0.01]** **0.017 (0.026)**	**−0.08 [−0.14, −0.007];** **0.033 (0.044)**	−0.08 [−0.14, −0.005] 0.036 (0.051)	−0.002 [−0.05, 0.05]; 0.936 (0.936)	0.02 [−0.03, 0.07] 0.492 (0.699)
**Frontal NACtx**	**−0.12 [−0.17, −0.07];** **< 0.0001 (0.0002)**	**−0.11 [−0.17, −0.06]** **0.0003 (0.001)**	**−0.09 [−0.16, −0.013]; 0.023 (0.037)**	−0.08 [−0.16, −0.007] 0.034 (0.051)	−0.03 [−0.03, 0.07]; 0.241 (0.887)	−0.02 [−0.07, 0.03] 0.399 (0.699)
**Temporal NACTx**	**−0.08 [−0.13, −0.03]** **0.005 (0.007)**	**−0.08 [−0.13, −0.02]** **0.006 (0.012)**	**−0.09 [−0.16, −0.02];** **0.010 (0.037)**	−0.08 [−0.16, −0.007] 0.034 (0.051)	0.02 [−0.03, 0.05]; 0.420 (0.887)	0.03 [−0.03, 0.09] 0.258 (0.699)
**Parietal NACtx**	−0.05 [−0.12, 0.01]; 0.1113 (0.129)	−0.05 [−0.12, 0.02] 0.191 (0.218)	−0.05 [−0.15, 0.04]; 0.249 (0.249)	−0.04 [−0.12, 0.05] 0.378 (0.432)	5 10^−3^ [−0.06, 0.07]; 0.872 (0.936)	0.01 [−0.05,0.07] 0.685 (0.783)
**Occipital NACtx**	−0.03 [−0.09, 0.03]; 0.323 (0.323)	0.01 [−0.06, 0.07] 0.807 (0.807)	−0.05 [−0.13, 0.03]; 0.188 (0.214)	−0.009 [−0.07, 0.05] 0.767 (0.767)	0.03 [−0.03, 0.09]; 0.308 (0.887)	0.05 [−0.004, 0.10] 0.074(0.591)
**Limbic NACtx**	**−0.07 [−0.12, −0.01]; 0.013 (0.018)**	−0.05 [−0.10, 0.003] 0.064 (0.085)	**−0.07 [−0.13, −0.01]; 0.021 (0.037)**	−0.07 [−0.14, −0.01] 0.021 (0.051)	0.01 [−0.04, 0.06]; 0.665 (0.887)	0.03 [−0.03, 0.08] 0.372 (0.699)
**Hippocampus NACtx**	**−0.12 [−0.18, −0.06];** **0.0002 (0.0007)**	**−0.12 [−0.18, −0.06]** **0.0003 (0.001)**	**−0.09 [−0.17, −0.02]; 0.018 (0.037)**	−0.10 [−0.17, −0.025] 0.012 (0.051)	−0.02 [−0.07, 0.03]; 0.452 (0.887)	−0.02 [−0.07, 0.03] 0.524 (0.699)
**Insular NACtx**	**−0.13 [−0.19, −0.07];** **0.0002 (0.0007)**	**−0.12 [−0.19, −0.05]** **0.001 (0.004)**	**−0.11 [−0.21, −0.02]; 0.016 (0.037)**	−0.09 [−0.18, −0.005] 0.038 (0.051)	−0.01 [−0.06, 0.04]; 0.663 (0.887)	−0.004 [−0.05, 0.05] 0.858 (0.858)

*Note:* Linear mixed effect models with mean MTsat in the NACtx region as dependent variable, binary disease group as primary predictor and random effect site (Oxford/Verona). Model 1 included age and sex as covariates; Model 2 adjusted for age, sex, and cortical thickness. Values report the between‐disease group difference coefficient with 95% CI and *p*‐value; the adjusted *p*‐value using FDR is shown in parentheses. Bold cells indicate results with adjusted *p*‐value < 0.05. MOGAD = myelin oligodendrocyte glycoprotein antibody‐associated disease; HC = healthy controls; NACtx = normal appearing cortex; T1/T2r z‐score = T1w‐toT2w signal ratio; MTR = Magnetization Transfer Ratio; MTsat = Magnetisation Transfer Saturation; CI = confidence interval; p _adj_ = *p*‐value corrected for multiple comparisons with false discovery rate.

#### 3.2.3 Normal‐Appearing Cortex Microstructural Damage and Clinical Outcomes in Cortical MOGAD Phenotype

**FIGURE 5:**
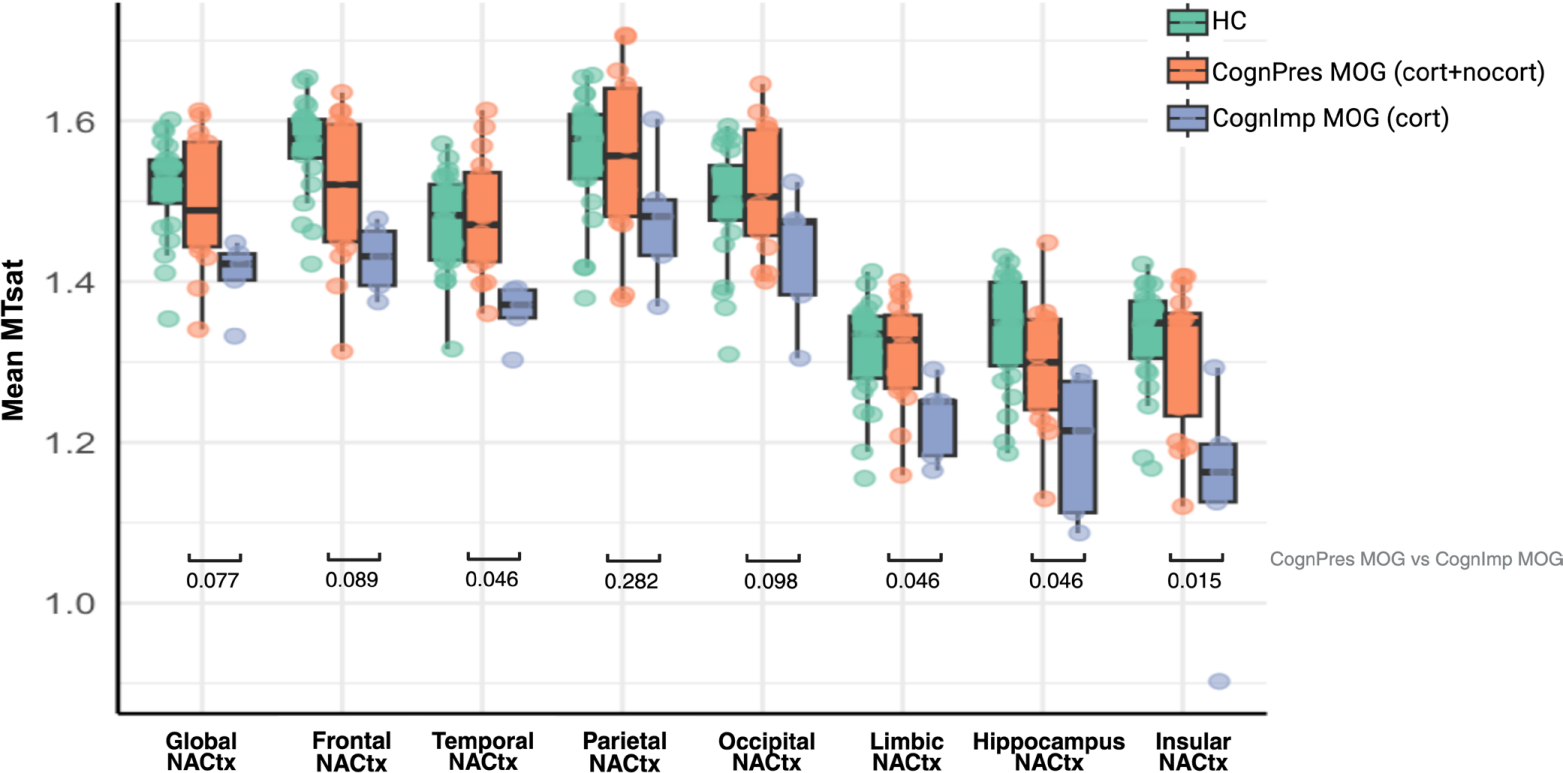
Regional normal‐appearing cortex MTsat differences among cognitive impaired MOGAD (cortical), cognitive preserved MOGAD (cortical and non‐cortical), and HC. Boxplots provide median and interquartile range of the mean MTsat within the regional normal appearing cortices (NACTx) of cognitive impaired MOGAD with cortical phenotype (CognImp MOG), of cognitive preserved MOGAD with non‐cortical and cortical phenotypes (CognPres MOG) and HCs. FDR‐corrected *p*‐value of linear mixed effect models 1 are provided dependent variable = mean regional NACtx MTsat; predictor variable = binary cognitive status (cognitive preserved vs. cognitive impaired); covariates = age, sex; random effect = MRI scanner site. P.u. = percentage units. Created in https://BioRender.com.

Among the 10 cortical‐MOGAD patients with MTI, five were cognitively impaired, and five were cognitively preserved (Figure 2). Cognitively impaired MOGAD showed a multidomain impairment profile, with the lowest mean z‐scores observed for PASAT‐2 (−2.33 ± 0.93), PASAT‐3 (−2.11 ± 0.96), and SDMT (−1.90 ± 1.74), followed by SRT‐D (−1.70 ± 1.19) and SRT‐LTS (−1.60 ± 0.62). Cognitively impaired patients had lower mean MTsat than cognitively preserved patients in global and regional NACtx (eTable 4). Compared with the overall cognitively preserved MOGAD patients with MTI (cortical and non‐cortical; *n* = 17), those with cortical MOGAD and cognitive deficit (*n* = 5) showed significantly lower Mtsat in temporal (β = −0.11, 95% CI –0.19 to −0.03, *p* = 0.012; p_adj = 0.046), limbic (β = −0.09, 95% CI –0.16 to −0.016, *p* = 0.019; p_adj = 0.046), hippocampal (β = −0.11, 95% CI –0.21 to −0.017, *p* = 0.023; p_adj = 0.046) and insular NACtx (β = −0.18, 95% CI –0.27 to −0.07, *p* = 0.002; p_adj = 0.015), which remained significant after additional adjustment for cortical thickness (Figure 5, eTable 5). When stratified by disability, six of 10 cortical‐MOGAD patients with MTI had moderate–severe disability (EDSS ≥ 3) and showed lower mean Mtsat than those with EDSS < 3 (*n* = 4) (eTable 4). When compared with MOGAD patients with EDSS < 3 and MTI (cortical and non‐cortical; *n* = 13), cortical MOGAD patients with moderate–severe disability (EDSS ≥ 3, *n* = 6) showed lower Mtsat in temporal and insular NACtx, but these differences were no longer significant after correction for multiple comparisons (eTable 5). Owing to the small subgroup sizes, these comparisons were considered exploratory.

## 4 Discussion

This study provides an in vivo assessment of normal‐appearing cortical microstructure in patients with MOGAD during remission using advanced quantitative MRI metrics—T1/T2r, MTR, and Mtsat—with established pathological specificity to myelin and neurite content validated in post‐mortem studies of MS.

Our findings reveal that (i) normal‐appearing cortex in MOGAD shows regional microstructural damage, most sensitively detected by MTsat; (ii) cortical MOGAD phenotype drives widespread NACtx MTsat changes, whereas non‐cortical MOGAD appears spared; (iii) within cortical MOGAD, lower MTsat appears to be associated with greater cognitive impairment and higher disability, although these relationships are explorative and may be partly mediated or confounded by age.

The primary analysis demonstrated significant MTsat reductions within the hippocampal, frontal and insular normal‐appearing cortex in the overall MOGAD cohort compared with healthy controls, with the hippocampal abnormality persisting after adjustment for cortical thickness, whereas T1/T2 ratio and MTR did not differ between groups. Taken together, these findings indicate that conventional structural and non‐quantitative imaging may substantially underestimate the burden of cortical injury in MOGAD and highlight MTsat as the most informative of the three qMRI measures. Consistent with this, a recent quantitative gradient‐recalled echo (qGRE) study reported reduced cortical R2t* in MOGAD relative to controls, although clinical phenotypes were not stratified and cortical–subcortical lesions were not excluded from the cortical grey‐matter masks, limiting direct comparison with the present work [29]. Among the parameters evaluated, MTsat showed the greatest ability to distinguish diseased from healthy cortex, in line with its technical advantages of correcting for B1 inhomogeneity and T1 relaxation effects that confound both MTR and the T1/T2‐weighted ratio [24]. A notable dissociation between MTR and MTsat was observed in limbic, hippocampal and insular cortex, where MTsat was globally lower than in other lobar regions, whilst MTR was preserved or elevated. This pattern reflects the intrinsic tissue properties of these regions rather than a measurement inconsistency: Limbic, hippocampal and insular cortices have intrinsically long T1, driven by low myelin density, low iron content and greater extracellular water fraction [30, 31, 32, 33, 34], which collectively inflate MTR whilst reducing MTsat through genuine macromolecular pool depletion [35]. MTsat, by simultaneously estimating T1 and macromolecular bound‐pool saturation, corrects for this confound and is therefore better positioned to capture subtle microstructural pathology in cortical regions that are architecturally complex and prone to partial‐volume effects [15]. Future multimodal quantitative MRI studies in larger cohorts will be important to determine which metrics, alone or in combination, offer the greatest translational potential for the clinical assessment of cortical pathology in MOGAD.

Patients with a history of cortical attacks showed significantly lower MTsat than healthy controls across global and multiple lobar NACtx regions, and lower MTsat than non‐cortical MOGAD, whereas the non‐cortical group did not differ from controls. These findings support the concept that, although inflammatory episodes in MOGAD are often clinically and radiologically transient [36], residual cortical injury can persist beyond the acute phase and tends to localise to regions inflamed during previous relapses [11]. At first sight, this may appear to conflict with animal work in which injection of patient‐derived MOG‐IgG into rats induces oedema and demyelination that largely resolves within two weeks [37], but experimental autoimmune encephalomyelitis models indicate that MOG‐IgG mainly amplifies central nervous system demyelination and is insufficient to reproduce the full clinicopathological spectrum of MOGAD without concomitant T‐cell‐mediated inflammation [38]. Localised acute meningeal inflammation and clusters of small intracortical lesions, together with residual cortico‐subcortical demyelination that may partially or completely normalise on conventional MRI, have been described in the limited MOGAD pathological series and provide a plausible substrate for the subtle qMRI abnormalities in normal‐appearing cortex observed in patients with cortical phenotypes in this study [7, 11]. Moreover, intrathecal synthesis of MOG‐IgG during acute phases has been linked to cortical presentations and may directly contribute to meningeal inflammation and cortical pathology, reinforcing the view that enduring cortical damage in MOGAD reflects the combined effects of antibody‐mediated and cellular immune mechanisms rather than transient antibody exposure alone [39, 40].

In exploratory analyses restricted to the cortical MOGAD subgroup, patients with cognitive impairment and those with moderate–severe disability (EDSS ≥ 3) showed lower mean MTsat within global and regional NACtx than cognitively preserved or less disabled patients, supporting a potential link between cortical microstructural damage, cognition and physical disability. In particular, cortical MOGAD patients with cognitive deficit exhibited reduced MTsat in temporal, limbic, hippocampal and insular NACtx compared with the overall cognitively preserved patients, and these differences remained after adjustment for cortical thickness, whereas MTsat reductions associated with higher EDSS were weaker and did not survive correction for multiple comparisons. This regional pattern is in keeping with the cognitive profile observed in our cohort, which was characterised mainly by deficits in processing speed, attention/working memory, and verbal learning/episodic memory. Taken together, these findings support a clinically relevant link between subtle cortical microstructural injury and cognitive impairment in cortical MOGAD, although the small subgroup size warrants cautious interpretation. These patterns may nonetheless be influenced by age, and it remains unclear whether older age primarily limits lesion repair detectable by quantitative MRI or whether age‐related cortical microstructural change interacts with disease‐related damage to produce the observed effects. Given the small subgroup sizes, these findings should be regarded as hypothesis‐generating, although they are consistent with MS studies in which microstructural abnormalities of normal‐appearing cortex have been associated with worse clinical and cognitive outcomes [41, 42, 43, 44]. Finally, the higher frequency of relapsing disease in cortical MOGAD, together with the absence of recurrent same‐region cortical relapses in most cases, may suggest that cortical involvement may occur during relapses without always being clinically or radiologically evident, a hypothesis supported by neuropathological evidence of small intracortical demyelinating lesions even in patients without dominant cortical manifestations [7], potentially contributing to subtle cumulative microstructural damage and impaired cortical repair over time.

### 4.1 Limitations

First, the cross‐sectional design precludes inferences about the temporal evolution of cortical microstructural changes following acute brain lesions, and longitudinal studies are needed to establish prognostic value. Second, although this represents one of the larger MOGAD qMRI series, the subset undergoing MTI was relatively small, reducing power for subgroup and multivariable analyses and meaning that some associations, particularly with cognition and disability, should be considered exploratory. Third, exploratory comparisons between treated and untreated patients did not show clear differences in MRI or cognitive measures; however, these analyses were limited by small and heterogeneous treatment subgroups, and treatment duration and type were not included as covariates in the main models. Larger, treatment‐stratified, longitudinal studies will be required to determine the impact of immunotherapy on cortical microstructural integrity in MOGAD. Finally, in the absence of direct histopathological correlation in MOGAD, the biological interpretation of MTsat and other qMRI abnormalities relies on extrapolation from MS and post‐mortem data, and dedicated clinico‐pathological studies will be required to confirm the underlying tissue substrates.

## 5 Conclusion

Our findings indicate that MOGAD with a cortical phenotype can lead to persistent, spatially localised cortical damage that remains undetectable on conventional imaging but is uncovered by advanced myelin‐sensitive quantitative techniques such as MTsat, and that such abnormalities are concordant with emerging links to cognitive and physical disability. Collectively, our findings advance the field by positioning myelin‐sensitive qMRI and MTsat as a candidate tool for quantifying cortical integrity and for capturing the residual impact of cortical relapses beyond what standard imaging reveals. Future longitudinal, multicentre studies integrating advanced multimodal MRI with pathological profiling should clarify the mechanisms driving grey‐matter vulnerability in MOGAD and establish which quantitative metrics have the greatest value as prognostic tools in routine practice.

## Author Contributions

V.C. Conception and design of the study, acquisition and analysis of data, drafting a significant portion of the manuscript or figures. A.T. Acquisition and analysis of data, drafting a significant portion of the manuscript or figures. S.M. Acquisition and analysis of data, drafting a significant portion of the manuscript or figures. N.D. Acquisition and analysis of data, drafting a significant portion of the manuscript or figures. M.T. Acquisition and analysis of data. S.Z. Acquisition and analysis of data. M.F. Acquisition and analysis of data. M.G.P. Acquisition and analysis of data. D.F. Acquisition and analysis of data. F.C. Acquisition and analysis of data. F.R. Acquisition and analysis of data. A.B. Acquisition and analysis of data. S.M. Acquisition and analysis of data. D.M. Acquisition and analysis of data. F.B.P. Acquisition and analysis of data. M.I.L. Acquisition and analysis of data. R.M. Drafting a significant portion of the manuscript. P.W. Acquisition and analysis of data, drafting a significant portion of the manuscript or figures. M.C. Design of the study, acquisition of data, drafting a significant portion of the manuscript. J.P. Conception and design of the study, acquisition and analysis of data, drafting a significant portion of the manuscript or figures. R.G. Conception and design of the study, acquisition and analysis of data, drafting a significant portion of the manuscript or figures.

## Funding

The authors have nothing to report.

## Conflicts of Interest

V. Camera received European Charcot Foundation and ECTRIMS fellowship grants, honoraria for speaking, and travel grants for scientific meetings from Roche, Janssen, BMS, Novartis, Alexion, and Amgen; received honoraria for advisory work from Novartis; and is a Member of the European Charcot Foundation young investigators/fellow community; A. Tamanti reports no disclosures; S. Messina received speaking honoraria from UCB and travel grants from Sanofi, UCB, and Merck, Alexion and Roche; N. Dall'Osto reports no disclosures; T. Maltempo reports no disclosures; S. Ziccardi reports no disclosures; M. Foschi served as scientific consultant for Roche and Novartis, and he received grants for travel and meeting attendance from Roche, Novartis, Biogen, Merck, Sanofi and Bristol‐Myers‐Squibb; M.G. Piscaglia reports no disclosures; D. Ferraro reports no disclosures; F. Crescenzo reports no disclosures; *F. Rossi* reports no disclosures; A. Bajrami reports no disclosures; S. Marangoni reports no disclosures. Dr Marastoni was supported by the GR‐2021‐12,373,041 grant from the Italian Ministry of Health. Prof Calabrese was supported by the RF‐2021‐12,373,319 grant from the Italian Ministry of Health. Dr Magliozzi was supported by a grant from the Italian MS Foundation (FISM 2023/R‐Single/038). #NEXTGENERATIONEU (NGEU) and funded by the Ministry of University and Research (MUR), National Recovery and Resilience Plan (NRRP), project MNESYS (PE0000006) – A Multiscale integrated approach to the study of the nervous system in health and disease (DN. 1553 11.10.2022). F.B. Pizzini reports no disclosures. M.I. Leite is funded by the NHS (Myasthenia and Related Disorders Service and National Specialised Commissioning Group for Neuromyelitis Optica, United Kingdom) and by the University of Oxford, United Kingdom; has been awarded research grants from the UK Association for Patients with Myasthenia (Myaware), Muscular Dystrophy Campaign (MDUK), and the University of Oxford; has received speaker honoraria and travel grants from UCB Pharma and Horizon Therapeutics; has received consultancy fees from UCB Pharma; and serves on scientific or educational advisory boards for UCB Pharma, Argenx, and Horizon Therapeutics; and on the steering committee for Horizon Therapeutics. R. Magliozzi: No disclosures. P. Waters is a named inventor on patents for antibody assays (WO/2010/046716) with royalties paid by Euroimmun AG and disease biomarker patents (WO2019211633A1, WO2022189788A1). He has received honoraria from Biogen Idec, Mereo Biopharma, Retrogenix, UBC, Euroimmun AG, UCB, F. Hoffmann La‐Roche, Forum for Indian Neurology Education (FINE) and Alexion; travel grants from the Guthy‐Jackson Charitable Foundation; and research funding from Euroimmun AG, the Sumaira Foundation and the Guthy‐Jackson Charitable Foundation. His work in the Oxford Autoimmune Neurology Diagnostic Laboratory is partly supported by the NHS Commissioning Service for NMOSD. He serves on the editorial boards of Neurology, Neuroimmunology & Neuroinflammation and the Journal of Clinical Neurology. M. Calabrese received honoraria for research or speaking and travel grants from Roche, Biogen Idec, Sanofi‐Genzyme, Novartis, and BMS; received grant research from the Italian Ministry of Health; and received honoraria for advisory work from Novartis, Roche, Merck, and BMS, Biogen. J. Palace has received support for scientific meetings and honoraria for advisory work from Merck Serono, Novartis, Chugai, Alexion, Roche, Medimmune, Amgen, Vitaccess, UCB, Mitsubishi, Amplo, and Janssen; has received grants from Alexion, Argenx, Clene, Roche, Medimmune, and Amplo Biotechnology; holds Patent ref. P37347WO and licence agreement Numares multimarker MS diagnostics Shares in AstraZeneca; her group has been awarded an ECTRIMS fellowship and a Sumaira Foundation grant to start later this year; a Charcot fellow worked in Oxford 2019–2021; she acknowledges partial funding to the trust by highly specialised services NHS England; is on the medical advisory boards of the Sumaira Foundation and MOG project charities; is a member of the Guthy‐Jackson Charitable Foundation; is on the board of the European Charcot Foundation; is a member of MAGNIMS and the UK NHSE IVIG Committee; is chairperson of the NHSE neuroimmunology patient pathway; has been an ECTRIMS council member on the educational committee since June 2023; is currently on the ABN advisory groups for MS and neuroinflammation; and was recently on a neuromuscular diseases advisory group.

## Supporting information


**Table e1:** Oxford and Verona brain 3 T MRI acquisition parameters.
3 T = 3Tesla; 3DMPRAGE = three‐dimentional Magnetisation‐Prepared Rapid Gradient‐Echo; 3DFLAIR = 3D Fluid‐Attenuated Inversion Recovery; 3DDIR = 3D Double Inversion Recovery; 2D/3D T2 TSE = two‐dimentiional/3D T2‐weighted Turbo Spin Echo; MTw = magnetisation transfer‐weighted; PDw = proton density‐weighted; T1w = T1‐weighted.

**Table e2:** Clinical and Imaging characteristics of MOGAD and HCs cohorts divided according to quantitative MRI acquisition protocols.
*Lesions with volume < 9 mm3 were labelled as zero volume. MOGAD = myelin oligodendrocyte glycoprotein antibody associated disease; HCs = healthy controls; SD = standard deviation; T1/T2r cohort = participants who underwent the acquisition protocol including 3DT1weighted and 3D/2D T2weigheted imaging for calculating T1w/T2w ratio z‐score metric; MTI cohort = participants who underwent the acquisition protocol including magnetisation transfer imaging to obtain magnetisation transfer ratio and magnetisation transfer saturation metrics; NA = not applicable.

**Table e3:** Clinical and Imaging characteristics of cortical, non‐cortical MOGAD and HCs cohorts with MTI acquisition.
MOGAD = myelin oligodendrocyte glycoprotein antibody associated disease; HCs = healthy controls; SD = standard deviation; 3DT1/T2w cohort = participants who underwent the acquisition protocol including 3DT1weighted and 3D/2D T2weigheted imaging for calculating T1w/T2w ratio z‐score metric; MTI cohort = participants who underwent the acquisition protocol including magnetisation transfer imaging to obtain magnetisation transfer ratio and magnetisation transfer saturation metrics; NA = not applicable.
*Lesions with volume < 9 mm3 were labelled as zero volume.
(a) cortical MOGAD vs. HCs *t*‐test *p*‐value < 0.05; (b) non‐cortical MOGAD vs. HCs *t*‐test *p*‐value < 0.05.

**Table e4:** Mean MTsat in normal‐appearing cortical regions among HC, cortical MOGAD patients with and without cognitive and physical disability.
MTsat = magnetisation transfer saturation; MTI = magnetisation transfer imaging; HC = healthy controls; MOGAD = myelin oligodendrocyte glycoprotein antibody‐associated disease; NACtx = normal appearing‐cortex; EDSS = Expanded Disability Status Scale; SD = standard deviation.

**Table e5:** Explorative linear mixed effect models evaluating the associations between mean MTsat in normal appearing cortices and clinical outcomes.
Linear mixed effect models with mean MTsat in the NACtx region as the dependent variable, disease group as the primary predictor and random effect site (Oxford/Verona). Model 1 included age and sex as covariates; Model 2 adjusted for age, sex, and cortical thickness. Values report the between‐disease group difference coefficient with 95% CI and *p*‐value; the adjusted *p*‐value is shown in parentheses. Bold cells indicate results with adjusted *p*‐value < 0.05. NACtx = normal appearing cortex on 3DFLAIR and 3D DIR sequences. *Significant also comparing cognitive impaired cortical MOGAD (*n* = 5) vs. cognitive preserved cortical MOGAD (*n* = 5).

## Data Availability

The data that support the findings of this study are available on request from the corresponding author. The data are not publicly available due to privacy or ethical restrictions.
