# Supplementary material for: Quantitative MRI Uncovers Subtle Cortical Damage in Myelin Oligodendrocyte Glycoprotein Antibody‐Associated Disease
Source: Ann Clin Transl Neurol. 2026 Jul 13:10.1002/acn3.70469. Online ahead of print. doi: 10.1002/acn3.70469 (PMC13394544; doi:10.1002/acn3.70469)
Supplement: Supplementary file 1 — Table e1: Oxford and Verona brain 3 T MRI acquisition parameters. 3 T = 3Tesla; 3DMPRAGE = three‐dimentional Magnetisation‐Prepared Rapid Gradient‐Echo; 3DFLAIR = 3D Fluid‐Attenuated Inversion Recovery; 3DDIR = 3D Double Inversion Recovery; 2D/3D T2 TSE = two‐dimentiional/3D T2‐weighted Turbo Spin Echo; MTw = magnetisation transfer‐weighted; PDw = proton density‐weighted; T1w = T1‐weighted. [file ACN3-9999-0-s005.docx]

**eTable 1: Oxford and Verona brain 3T MRI acquisition parameters**

|  | **3DMPRAGE** | **3DFLAIR** | **3DDIR** | **2DT2 TSE*** |  |
| --- | --- | --- | --- | --- | --- |
| **Oxford - 3T Siemens MAGNETOM® Prisma** |  |  |  |  |  |
| Repetition Time (TR) | 2040 ms | 5000 ms | 7500 ms | 5690ms |  |
| Echo Time (TE) | 4.7 ms | 397ms | 380 ms | 71ms |  |
| Inversion Time (TI) | 900 ms | 1800 ms | 450 ms/3000 ms |  |  |
| Flip Angle/refocusing control | 8° | 120° | 40° | 120° |  |
| Voxel size | 1x1x1mm^3^ | 1x1x1mm^3^ | 1x1x1mm^3^ | 0.5x0.5x3mm^3^ |  |
| Field Of View (FOV) | 174x192x192 | 256x256x192 | 256x256x176 | 220x220x144 |  |
| Number of Signals Averaged (NSA) | 1 | 1 | 3 | 1 |  |
| Acquisition time | 5:56 min | 5:52 min | 7:15 min | 2:07 |  |
| Sequence specific parameters |  |  |  |  |  |
| **Verona – 3T Philips ELITION-S** | **3DMPRAGE** | **3DFLAIR** | **3DDIR** | **3DT2 TSE*** | **3DMTw/PDw/T1w** |
| Repetition Time (TR) | 8.4 ms | 8000ms | 5500 ms | 3000ms | 25/25/11 |
| Echo Time (TE) | 3.8 ms | 380ms | 315 ms | 260ms | 3.7ms |
| Inversion Time (TI) | 1050 ms | 2356 ms | 2550 ms/450 ms |  |  |
| Flip Angle/refocusing control | 8° | 40° | 40° | 45° | 5°/5°/15° |
| Voxel size | 1x1x1mm^3^ | 1x1x1mm^3^ | 1x1x1mm^3^ | 1x1x1mm^3^ | 1x1x1mm^3^ |
| Field Of View (FOV) | 240x240x180 | 240x240x180 | 240x240x168 | 240x240x180 | 240x240x180 |
| Number of Signals Averaged (NSA) | 1 | 1 | 3 | 1 | 1 |
| Acquisition time | 3:29 min | 3:52 min | 6:41 min | 3:21 min | 10:08 min |
| Magnetization transfer pre-pulse |  |  |  |  | MT off-resonance/no/no |

Legend: 3T=3Tesla; 3DMPRAGE=three-dimentional Magnetization-Prepared Rapid Gradient-Echo; 3DFLAIR= 3D Fluid-Attenuated Inversion Recovery; 3DDIR= 3D Double Inversion Recovery; 2D/3D T2 TSE= two-dimentiional/3D T2-weighted Turbo Spin Echo; MTw = magnetization transfer-weighted; PDw = proton density-weighted; T1w = T1-weighted.
