# Supplementary material for: Quantitative MRI Uncovers Subtle Cortical Damage in Myelin Oligodendrocyte Glycoprotein Antibody‐Associated Disease
Source: Ann Clin Transl Neurol. 2026 Jul 13:10.1002/acn3.70469. Online ahead of print. doi: 10.1002/acn3.70469 (PMC13394544; doi:10.1002/acn3.70469)
Supplement: Supplementary file 2 — Table e2: Clinical and Imaging characteristics of MOGAD and HCs cohorts divided according to quantitative MRI acquisition protocols. *Lesions with volume < 9 mm3 were labelled as zero volume. MOGAD = myelin oligodendrocyte glycoprotein antibody associated disease; HCs = healthy controls; SD = standard deviation; T1/T2r cohort = participants who underwent the acquisition protocol including 3DT1weighted and 3D/2D T2weigheted imaging for calculating T1w/T2w ratio z‐score metric; MTI cohort = participants who underwent the acquisition protocol including magnetisation transfer imaging to obtain magnetisation transfer ratio and magnetisation transfer saturation metrics; NA = not applicable. [file ACN3-9999-0-s003.docx]

**eTable 2: Clinical and Imaging characteristics of MOGAD and HCs cohorts divided according to quantitative MRI acquisition protocols**

|  | **MOGAD**  **T1/T2r cohort**  **n=42** | **HC**  **T1/T2r cohort**  **n=42** | **p-value** | **MOGAD**  **MTI cohort**  **n=22** | **HC**  **MTI cohort**  **n=24** | **p-value** |
| --- | --- | --- | --- | --- | --- | --- |
| **Clinical MRI features** |  |  |  |  |  |  |
| Age, mean ± SD | 43.20 ±12.60 | 42.10±12.70 | 0.699 | 44.50±13.9 | 45.00±12.30 | 0.907 |
| Sex (F/M) | 23/19 | 26/16 | 0.658 | 13/9 | 16/8 | 0.761 |
| Disease course:  -Monophasic, n (%)  -Relapsing, n (%) | 14 (33.30)  28 (66.70) | NA  NA | NA  NA | 8 (36.4)  14 (63.6) | NA  NA | NA  NA |
| **Baseline MRI features** |  |  |  |  |  |  |
| Cortical lesions, median (range)   - Count - Volume (mm^3^)* | 0 (0-10)  0 (0-946) | NA  NA | NA  NA | 0 (0-10)  0 (0-946) | NA  NA | NA  NA |
| White matter lesions, median (range)   - Count - Volume (mm^3^)* | 1 (0-43)  46 (0-43030) | NA  NA | NA  NA | 3.5 (0-43)  215 (0-43030) | NA  NA | NA  NA |
| Deep grey matter lesions, median (range)   - Count - Volume (mm^3^)* | 0 (0-5)  0 (0-407) | NA  NA | NA  NA | 0 (0-5)  0 (0-407) | NA  NA | NA  NA |
| Cortical thickness, mean ± SD   - Global (mm^2^) - Frontal (mm^2^) - Temporal (mm^2^) - Parietal (mm^2^) - Occipital (mm^2^) - Limbic (mm^2^) - Hippocampus (mm^2^) - Insula (mm^2^) | 2.79 ± 0.27  2.62 ± 0.26  3.35 ± 0.32  2.36 ± 0.30  2.65 ± 0.38  3.29 ±0.25  2.99 ± 0.22  3.65 ± 0.54 | 2.96 ± 0.19  2.79 ± 0.21  3.59 ± 0.27  2.44 ± 0.22  2.80 ± 0.29  3.48 ± 0.22  2.96 ± 0.26  3.97 ± 0.45 | 0.001  0.001  <0.001  0.166  0.041  <0.001  0.580  0.004 | 2.76 ± 0.20  2.63 ± 0.21  3.29 ± 0.29  2.28 ± 0.16  2.56 ± 0.30  3.36 ±0.24  3.00 ± 0.26  3.83 ± 0.50 | 2.91 ± 0.17  2.76 ± 0.19  3.50 ± 0.20  2.34 ± 0.17  2.78 ± 0.30  3.55 ± 0.21  2.98 ± 0.23  4.05 ± 0.44 | 0.007  0.031  0.006  0.225  0.018  0.007  0.789  0.132 |
| T1/T2r z-score, mean ± SD   - Global - Frontal - Temporal - Parietal - Occipital - Limbic - Hippocampus - Insula | -0.06 ±0.81  0.01 ±1.12  -0.09 ±0.73  0.23 ±1.24  0.15 ±1.09  -0.03 ±1.26  -0.16 ±1.33  -0.16 ±1.13 | 0.00 ±0.98  0.00 ±0.98  0.00 ±0.98  0.00 ±0.98  0.00 ±0.98  0.00 ±0.98  0.00 ±0.98  0.00 ±0.98 | 0.766  0.953  0.624  0.374  0.545  0.918  0.540  0.527 | NA  NA  NA  NA  NA  NA  NA  NA | NA  NA  NA  NA  NA  NA  NA  NA | NA  NA  NA  NA  NA  NA  NA  NA |
| MTR, mean ± SD   - Global - Frontal - Temporal - Parietal - Occipital - Limbic - Hippocampus - Insula | NA  NA  NA  NA  NA  NA  NA  NA | NA  NA  NA  NA  NA  NA  NA  NA | NA  NA  NA  NA  NA  NA  NA  NA | 39.60 ±1.63  38.36 ±1.74  39.30 ±1.78  39.76 ±1.71  41.95 ±1.53  41.77 ±1.84  42.14 ±2.40  39.23 ±3.06 | 39.39 ±1.61  38.20 ±1.45  39.49 ±1.72  39.18 ±1.84  41.56 ±1.93  41.63 ±1.68  43.07 ±2.04  40.17 ±1.85 | 0.669  0.731  0.711  0.280  0.452  0.792  0.171  0.229 |
| MTsat, mean ± SD   - Global - Frontal - Temporal - Parietal - Occipital - Limbic - Hippocampus - Insula | NA  NA  NA  NA  NA  NA  NA  NA | NA  NA  NA  NA  NA  NA  NA  NA | NA  NA  NA  NA  NA  NA  NA  NA | 1.48 ±0.08  1.49 ±0.09  1.45±0.08  1.53 ±0.10  1.50 ±0.09  1.29 ±0.07  1.27 ±0.09  1.26 ±0.12 | 1.52 ±0.06  1.57 ±0.06  1.48 ±0.06  1.56 ±0.07  1.50 ±0.07  1.32 ±0.06  1.34 ±0.07  1.33 ±0.07 | 0.073  0.004  0.284  0.376  0.968  0.234  0.008  0.030 |

Legend: *Lesions with volume < 9mm3 were labelled as zero volume. MOGAD= myelin oligodendrocyte glycoprotein antibody associated disease; HCs= healthy controls; SD= standard deviation; T1/T2r cohort= participants who underwent the acquisition protocol including 3DT1weighted and 3D/2D T2weigheted imaging for calculating T1w/T2w ratio z-score metric; MTI cohort= participants who underwent the acquisition protocol including magnetization transfer imaging to obtain magnetization transfer ratio and magnetization transfer saturation metrics; NA= not applicable
