# Supplementary material for: Quantitative MRI Uncovers Subtle Cortical Damage in Myelin Oligodendrocyte Glycoprotein Antibody‐Associated Disease
Source: Ann Clin Transl Neurol. 2026 Jul 13:10.1002/acn3.70469. Online ahead of print. doi: 10.1002/acn3.70469 (PMC13394544; doi:10.1002/acn3.70469)
Supplement: Supplementary file 3 — Table e3: Clinical and Imaging characteristics of cortical, non‐cortical MOGAD and HCs cohorts with MTI acquisition. MOGAD = myelin oligodendrocyte glycoprotein antibody associated disease; HCs = healthy controls; SD = standard deviation; 3DT1/T2w cohort = participants who underwent the acquisition protocol including 3DT1weighted and 3D/2D T2weigheted imaging for calculating T1w/T2w ratio z‐score metric; MTI cohort = participants who underwent the acquisition protocol including magnetisation transfer imaging to obtain magnetisation transfer ratio and magnetisation transfer saturation metrics; NA = not applicable. *Lesions with volume < 9 mm3 were labelled as zero volume. (a) cortical MOGAD vs. HCs t‐test p‐value < 0.05; (b) non‐cortical MOGAD vs. HCs t‐test p‐value < 0.05. [file ACN3-9999-0-s001.docx]

**eTable 3: Clinical and Imaging characteristics of cortical, non-cortical MOGAD and HCs cohorts with MTI acquisition**

|  | **Cortical MOGAD**  **n=10** | **Non-cortical MOGAD**  **n=12** | **HC**  **n=24** | **p-value** |
| --- | --- | --- | --- | --- |
| **Clinical MRI features** |  |  |  |  |
| Age, mean ± SD | 46.6 ± 16.9 | 42.8 ±11.3 | 45.0 ± 12.30 | 0.788 |
| Sex (F/M) | 6/4 | 7/5 | 16/8 | 0.761 |
| Disease course:  -Monophasic, n (%)  -Relapsing, n (%) | 1 (10)  9 (90) | 7 (58.3)  5 (41.7) | NA  NA | 0.031 |
| **Baseline MRI features** |  |  |  |  |
| Cortical lesions, median (range)   - Count - Volume (mm^3^)* | 0 (0-10)  0 (0-946) | 0 (0-0)  0 (0-0) | NA  NA | 0.053  0.053 |
| White matter lesions, median (range)   - Count - Volume (mm^3^)* | 6 (0-21)  1055.5 (0-43030) | 2 (0-43)  127.5 (0-32875) | NA  NA | 0.712  0.366 |
| Deep grey matter lesions, median (range)   - Count - Volume (mm^3^)* | 0 (0-5)  0 (0-407) | 0 (0-2)  0 (0-89) | NA  NA | 0.895  0.895 |
| Cortical thickness, mean ± SD   - Global (mm^2^) - Frontal (mm^2^) - Temporal (mm^2^) - Parietal (mm^2^) - Occipital (mm^2^) - Limbic (mm^2^) - Hippocampus (mm^2^) - Insula (mm^2^) | 2.72 ± 0.25^a^  2.57 ± 0.24^a^  3.33 ± 0.38  2.23 ± 0.15  2.42 ± 0.33 ^a^  3.36 ± 0.32  2.97 ± 0.14  3.64 ± 0.46 | 2.79 ± 0.15  2.67 ± 0.17  3.25 ± 0.18 ^b^  2.32 ± 0.17  2.67 ± 0.24  3.35 ± 0.17 ^b^  3.02 ± 0.33  3.99 ± 0.50 | 2.91 ± 0.17  2.76 ± 0.19  3.50 ± 0.20  2.34 ± 0.17  2.78 ± 0.30  3.55 ± 0.21  2.98 ± 0.23  4.05 ± 0.44 | 0.018  0.046  0.016  0.242  0.009  0.026  0.873  0.069 |

MOGAD= myelin oligodendrocyte glycoprotein antibody associated disease; HCs= healthy controls; SD= standard deviation; 3DT1/T2w cohort= participants who underwent the acquisition protocol including 3DT1weighted and 3D/2D T2weigheted imaging for calculating T1w/T2w ratio z-score metric; MTI cohort= participants who underwent the acquisition protocol including magnetization transfer imaging to obtain magnetization transfer ratio and magnetization transfer saturation metrics; NA= not applicable

*Lesions with volume < 9mm3 were labelled as zero volume.

^a^ cortical MOGAD versus HCs t-test p-value <0.05; ^b^non-cortcal MOGAD versus HCs t-test p-value <0.05
