# Supplementary material for: Quantitative MRI Uncovers Subtle Cortical Damage in Myelin Oligodendrocyte Glycoprotein Antibody‐Associated Disease
Source: Ann Clin Transl Neurol. 2026 Jul 13:10.1002/acn3.70469. Online ahead of print. doi: 10.1002/acn3.70469 (PMC13394544; doi:10.1002/acn3.70469)
Supplement: Supplementary file 4 — Table e4: Mean MTsat in normal‐appearing cortical regions among HC, cortical MOGAD patients with and without cognitive and physical disability. MTsat = magnetisation transfer saturation; MTI = magnetisation transfer imaging; HC = healthy controls; MOGAD = myelin oligodendrocyte glycoprotein antibody‐associated disease; NACtx = normal appearing‐cortex; EDSS = Expanded Disability Status Scale; SD = standard deviation. [file ACN3-9999-0-s004.docx]

**eTable 4: Mean MTsat in normal‑appearing cortical regions among HC, cortical MOGAD patients with and without cognitive and physical disability**

|  | **HC with MTI** | **Cortical MOGAD with MTI**  **n=10** | | | |
| --- | --- | --- | --- | --- | --- |
|  | **n=24** | **Cognitive preserved**  **n=5** | **Cognitive deficit**  **n=5** | **EDSS <3**  **n=4** | **EDSS ≥ 3**  **n=6** |
|  | **MTsat mean ± SD** | **MTsat mean ± SD** | **MTsat mean ± SD** | **MTsat mean ± SD** | **MTsat mean ± SD** |
| **Global NACtx** | 1.518 ± 0.060 | 1.40±0.11 | 1.31±0.04 | 1.46±0.11 | 1.30±0.19 |
| **Frontal NACtx** | 1.568 ± 0.058 | 1.46±0.18 | 1.43±0.19 | 1.46±0.12 | 1.44±0.05 |
| **Temporal NACTx** | 1.476 ± 0.061 | 1.44±0.10 | 1.36±0.04 | 1.44±0.12 | 1.38±0.04 |
| **Parietal NACtx** | 1.559 ± 0.075 | 1.53±0.12 | 1.48±0.09 | 1.54±0.14 | 1.48±0.08 |
| **Occipital NACtx** | 1.497 ± 0.073 | 1.50±0.08 | 1.43±0.09 | 1.52±0.074 | 1.43±0.08 |
| **Limbic NACtx** | 1.316 ± 0.064 | 1.27±0.08 | 1.23±0.05 | 1.27±0.09 | 1.24±0.05 |
| **Hippocampus NACtx** | 1.339 ± 0.070 | 1.25±0.09 | 1.20±0.09 | 1.24±0.09 | 1.21±0.09 |
| **Insular NACtx** | 1.332 ± 0.065 | 1.25±0.07 | 1.14±0.15 | 1.27±0.11 | 1.14±0.13 |
