# Supplementary material for: Quantitative MRI Uncovers Subtle Cortical Damage in Myelin Oligodendrocyte Glycoprotein Antibody‐Associated Disease
Source: Ann Clin Transl Neurol. 2026 Jul 13:10.1002/acn3.70469. Online ahead of print. doi: 10.1002/acn3.70469 (PMC13394544; doi:10.1002/acn3.70469)
Supplement: Supplementary file 5 — Table e5: Explorative linear mixed effect models evaluating the associations between mean MTsat in normal appearing cortices and clinical outcomes. Linear mixed effect models with mean MTsat in the NACtx region as the dependent variable, disease group as the primary predictor and random effect site (Oxford/Verona). Model 1 included age and sex as covariates; Model 2 adjusted for age, sex, and cortical thickness. Values report the between‐disease group difference coefficient with 95% CI and p‐value; the adjusted p‐value is shown in parentheses. Bold cells indicate results with adjusted p‐value < 0.05. NACtx = normal appearing cortex on 3DFLAIR and 3D DIR sequences. *Significant also comparing cognitive impaired cortical MOGAD (n = 5) vs. cognitive preserved cortical MOGAD (n = 5). [file ACN3-9999-0-s002.docx]

**eTable5: Explorative linear mixed effect models evaluating the associations between mean MTsat in normal appearing cortices and clinical outcomes**

|  | **Cogn Impaired Cortical MOGAD (n=5) vs Cogn Preserved MOGAD (n=17; ref)** | | **EDSS ≥3 Cortical MOGAD (n=6) vs EDSS <3 MOGAD (n=13; ref)** | |
| --- | --- | --- | --- | --- |
| **MTsat** | **Model 1**  **β [95% CI]**  **p-value (p _adj_)** | **Model 2**  **β [95% CI]**  **p-value (p _adj_)** | **Model 1**  **β [95% CI]**  **p-value (p _adj_)** | **Model 2**  **β [95% CI]**  **p-value (p _adj_)** |
| **Global NACtx** | -0.09 [-0.17, -0.001]  0.048 (0.077) | -0.09 [-0.18, 0.01]  0.079 (0.127) | -0.06 [-0.16, 0.037]  0.202 (0.324) | -0.06 [-0.17, 0.039]  0.194 (0.388) |
| **Frontal NACtx** | -0.9 [-0.19, 0.007]  0.060 (0.089) | -0.08 [-0.18, 0.02]  0.113 (0.150) | -0.05 [-0.17, 0.06]  0.346 (0.395) | -0.05 [-0.17, 0.07]  0.399 (0.531) |
| **Temporal NACTx** | **-0.11 [-0.19, -0.03]**  **0.012 (0.046)** | **-0.11 [-0.19, -0.03]**  **0.008 (0.033)** | -0.01 [-0.199, -0.005]  0.040 (0.187) | -0.09 [-0.17, -0.003]  0.043 (0.171) |
| **Parietal NACtx** | -0.06 [-0.18, 0.06]  0.282 (0.282) | -0.03 [-0.14, 0.09]  0.642 (0.733) | -0.04 [-0.17, 0.109]  0.548 (0.548) | -0.02 [-0.14, 0.11]  0.798 (0.798) |
| **Occipital NACtx** | -0.08 [-0.17. 0.12]  0.086 (0.098) | -0.006 [0.09, 0.07]  0.873 (0.873) | -0.07 [-0.16, 0.03]  0.167 (0.323) | 0.02 [-0.07, 0.11]  0.689 (0.789) |
| **Limbic NACtx** | **-0.09 [-0.16, -0.016]**  **0.019 (0.046)** | **-0.09 [-0.17, -0.019]**  **0.017 (0.034)** | -0.05 [-0.14, 0.04]  0.246 (0.327) | -0.051 [-0.14, 0.04]  0.257 (0.412) |
| **Hippocampus NACtx** | **-0.11 [-0.21, -0.17]**  **0.023 (0.046)** | **-0.12 [-0.21, -0.03]**  **0.016 (0.034)** | -0.07 [-0.18, 0.03]  0.142 (0.323) | -0.07 [-0.18, 0.035]  0.166 (0.388) |
| **Insular NACtx** | **-0.18 [-0.27, -0.07]**  **0.002 (0.015)** | **-0.16 [-0.25, -0.06]**  **0.003 (0.026) *** | -0.13 [-0.25, -0.002]  0.047 (0.187) | -0.13 [-0.24, -0.01]  0.037 (0.171) |

Linear mixed effect models with mean MTsat in NACtx region as dependent variable, disease group as primary predictor and random effect site (Oxford/Verona). Model 1 included age and sex as covariates; Model 2 adjusted for age, sex, and cortical thickness. Values report the between-disease group difference coefficient with 95% CI and p-value; the adjusted p-value is shown in parentheses. Bold cells indicate results with adjusted p-value < 0.05. NACtx= normal appearing cortex on 3DFLAIR and 3D DIR sequences

*Significant also comparing cognitive impaired cortical MOGAD (n=5) versus cognitive preserved cortical MOGAD (n=5)
